# Dépistage des maladies cardiovasculaires chez des étudiants de l'Université de Douala et influence des activités physiques et sportives

**Published:** 2012-04-24

**Authors:** Marielle Epacka Ewane, Samuel Honoré Mandengue, Eugene Belle Priso, Stéphane Moumbe Tamba, Ahmadou, André Bita Fouda

**Affiliations:** 1 Hôpital Général de Douala, Cameroun; 2 Université de Douala, Cameroun

**Keywords:** Etudiants, maladies cardiovasculaires, obésité, hypertension artérielle, diabète, activités physiques, Cameroun

## Abstract

**Introduction:**

Les maladies cardiovasculaires (MCV) constituent l'une des principales causes de mortalité dans les pays en développement. Le dépistage de ces dernières chez des jeunes est un défi dans la lutte contre leur expansion. Le but de cette étude était de dépister ces maladies au sein d'une population jeunes d’étudiants camerounais.

**Methodes:**

Deux mille six cent cinquante-huit étudiants de l'Université de Douala (23,6 ± 2,9 ans, sex-ratio H/F = 0,9) ont en Avril - Mai 2011 participé à une campagne de dépistage gratuit du diabète, de l'hypertension artérielle (HTA) et de l'obésité. Ils ont également été soumis à une d'enquête évaluant leur niveau en activités physiques et sportives (APS).

**Resultats:**

12,7% des participants avaient une pression artérielle (PA) ≥ 140/90 mmHg, 3,6% étaient obèses et 0,9% avaient une glycémie ≥1,26 g/L. Des corrélations ont été trouvées entre certains facteurs de risque (diabète, hypertension et obésité) et le niveau académique d'une part (r =0,366; p < 0,0001) et le temps passé devant la télévision d'autres part (r = 0,411; p < 0,0001). L‘APS était inversement corrélée à l‘âge (r =-0,015; p < 0,0001) et au temps passé devant la télévision (r = -0,059; p = 0,002).

**Conclusion:**

La présence des MCV et leurs facteurs de risque mis en évidence dans cette étude réalisée en milieu estudiantin camerounais interpelle à une prévention et une éducation dans la lutte contre ces dernières.

## Introduction

Le fardeau des maladies cardiovasculaires (MCV) dans le monde est énorme et croissant, et la majorité des personnes affectées se trouvent dans les pays émergents [1, 2]. Selon l'Organisation Mondiale de la Santé (OMS), 9,2% des décès en 2005 dans la région africaine étaient dus aux MCV [3] et en 2007, ce taux est passé à 20% [4]. La modernisation, la sédentarisation, et l'inactivité physique des populations surtout urbaines favorisent l'obésité et l'installation accrue de ces MCV [5, 6]. Au Cameroun, selon le Ministère de la Santé Publique, 25% de la population souffre d'hypertension artérielle. Une étude menée au Cameroun en 1998 a trouvé une prévalence d'hypertension artérielle (HTA) de 18,5% chez les hommes et 12,6% chez les femmes [7]. L'existence d'un diabète, d'une HTA, d'une obésité ou la diminution du temps consacré aux APS au profit du temps passé devant la télé peut favoriser l'apparition des complications cardio-vasculaires (maladies coronariennes, insuffisance cardiaque, accidents vasculaires cérébraux) [8–13]. Beaucoup d’études ont surtout porté sur les personnes âgées qui généralement, présentent un risque cardiovasculaire plus élevé [14], peu d’études à notre connaissance se sont appesanties sur la prévalence et l'impact des MCV chez une population constituée de sujets jeunes et plus particulièrement des étudiants camerounais. Compte tenu de la place qu'occupent les étudiants dans le devenir d'une nation, la présente étude a été menée sur cette population particulière dans le but de déterminer les prévalences du diabète, HTA et obésité et trouver des corrélations éventuelles entre ces maladies et les facteurs de risque dans le contexte de pays pauvre. Par ailleurs, étant donné la place avérée des effets positifs des APS dans la prévention de ces maladies et leurs facteurs de risque, une enquête évaluant le niveau de pratique de ces APS a été conduite.

## Méthodes

### Participants

En Avril-Mai 2011, 2696 étudiants des deux genres de l'Université de Douala ont volontairement participé à une campagne de dépistage gratuit des maladies cardiovasculaires (diabète, hypertension et obésité) et ont également rempli le questionnaire de Ricci et Gagnon [15] évaluant le niveau des activités physiques et sportives (APS). L’échantillonnage était aléatoire simple. Les étudiants âgés de plus de 35 ans ont été exclus de notre étude à cause de la susceptibilité aux risques cardiovasculaires plus accru à partir de cet âge. L’échantillon définitif était finalement constitué de 2658 étudiants. L’étude a reçu l'autorisation du Ministère de la santé publique et s'est conformée au code éthique de la Déclaration de Helsinki de 1975

### Méthodes

Le dépistage était réalisé à jeun depuis 12 heures au moins et comprenait l'identification du sujet à l'aide d'une fiche individuelle et la collecte d'antécédents personnels, familiaux (parents diabétiques, hypertendus ou obèses) et des habitudes toxiques (consommation d'alcool et tabac). La taille des sujets pieds nus, en position debout, ainsi que leur poids, étaient mesurés à l'aide d'un ensemble toise plus pèse-personne de marque DETECTO (Webb City, MO USA). L'indice de masse corporelle (IMC) était calculé suivant la formule IMC = poids (kg)/(taille(m))^2^ et a permis de définir le surpoids pour IMC ≥ 25 et < 30 Kg/m^2^ et l'obésité pour IMC > 30Kg/m^2^ [16]. Le tour de taille et le tour de hanche étaient mesurés à l'aide d'un mètre ruban; et le rapport tour taille sur tour de hanche (T/H) calculé. Les paramètres physiologiques (pression artérielle et fréquence cardiaque) étaient mesurés au repos depuis 15 min au moins, sur le bras gauche du sujet assis à l'aide d'un tensiomètre électronique à brassard de marque OMRON M3 Plus (Health Care CO. Ltd, Kyoto, Japon) homologué par l OMS. Une PA ≥ 140/90 mmHg [17] était considérée comme pression artérielle supérieure à la normale (PASN), étant donné qu une seule mesure ne peut pas permettre de déclarer un sujet hypertendu. Les sujets présentant une telle pression artérielle étaient invités à un contrôle le lendemain et une fois par semaine pendant un mois, avant que l'hypertension ne soit confirmée. La glycémie capillaire était mesurée à partir d'une goutte de sang prélevé sur l'index grâce au glucomètre électronique de marque ACCU-CHEK AVIVA (Roche Group Laboratory Equipment 207 W, USA) et les sujets qui présentaient une glycémie ≥ 1,26 g/L [18] étaient supposés diabétiques et invités à faire un contrôle une semaine plus tard avant que le diagnostic ne soit confirmé. A la fin de la mesure des paramètres, il a été proposé aux participants de répondre au questionnaire de Ricci et Gagnon [15] afin d’évaluer leur niveau d'APS par calcul du score obtenu. Ce questionnaire classe les sujets en inactifs, actifs et très actifs suivant des scores respectifs: < 15 points; entre 15 et 32 points; et > 32 points.

### Analyses statistiques

Les analyses statistiques ont été faites avec le logiciel Statview version 5.0 (SAS Institute Inc., Cary, NC). Les résultats sont donnés en moyenne ± DS. La statistique descriptive a permis de déterminer les prévalences ainsi que la répartition en fonction du sexe et de l’âge des maladies diagnostiquées. L'analyse statistique a utilisé pour la comparaison des moyennes et des proportions le *t*-test compte tenu de la taille de l’échantillon. Des analyses bivariées visant à établir des corrélations entre différents facteurs ont été réalisées grâce au test de Spearman. Les résultats ont été considérés significatifs à partir de p < 0,05.

## Résultats

Les sujets étaient répartis en 1254 hommes (47,2%) et 1404 femmes (52,8%) soit un sex-ratio de 0,9 avec une moyenne d’âge de 23,6 ± 2,9 ans (Tableau 1).

**Table 1:** Paramètres anthropométriques, physiologiques et métaboliques des étudiants

Paramètres	H (n = 1254)	F (n = 1404)	p
**Age (ans)**	23,9 ± 2,9	23,2 ± 2,8^ds^	< 0,0001
**Taille (m)**	1,73 ± 0,08	1,63 ± 0,06 ^ds^	< 0,0001
**Poids (kg)**	68,0 ± 10,1	61,5 ± 10,5 ^ds^	< 0,0001
**Tour de taille (cm)**	76,5± 7,6	76,6 ± 8,7	0,07
**Tour de hanche (cm)**	91,6 ± 9,6	93,9 ±9,5 ^ds^	< 0,0001
**T/H**	0,84 ± 0,07	0,82 ± 0,07 ^ds^	< 0,0001
**IMC (Kg/m**^**2**^**)**	22,7 ± 3,8	23,3 ± 3,8 ^ds^	0,01
**PAS (mmHg)**	126,4 ± 11,7	117,5 ± 11,7 ^ds^	< 0,0001
**PAD (mmHg)**	76,7 ± 9,4	74,2 ± 9,4 ^ds^	< 0,0001
**FC (bpm)**	69 ± 11	79 ± 11 ^ds^	< 0,0001
**Glycémie (g/L)**	0,86 ± 0,13	0,89 ± 0,13 ^ds^	0,0002

H: hommes F: femmes ds: différence significative

Le surpoids et l'obésité étaient respectivement retrouvés chez 19,4% et 3,6% de l’échantillon. L'obésité était plus fréquente chez les femmes que chez les hommes (5,8% vs 1,1%, p < 0,0001). L'IMC était respectivement corrélé au T/H (r = 0,137; p < 0,0001); au tour de hanche (r = 0,646; p < 0,0001), et à l’âge (r = 0,129; p = 0,0001). Trois cent trente-sept étudiants (soit 12,7%) avaient une PASN avec une prédominance masculine (8,5% vs 4,2%, p < 0,0001) tandis que 24 (soit 0,9%) avaient une glycémie ≥ 1,26 g/L les femmes ayant une prévalence plus élevée que les hommes (0,8% vs 0,1%, p = 0,001) Le Tableau 2 donne la répartition des différentes maladies cardiovasculaires suivant les tranches d’âge. hypertension et obésité et le niveau académique d'une part (r = 0,366; p < 0,0001) et avec le temps passé devant la télévision d'autre part (r = 0,411; p < 0,0001). Des corrélations positives ont été trouvées entre la glycémie et respectivement l'IMC (r = 0,158; < 0,0001), le T/H (r = 0,061; p = 0,001), l'hypertension une PASN(r = 0,357; p < 0,0001) et l'obésité (r = 0,481; p < 0,0001). Le Tableau 3 montre la répartition du niveau d'activité physique dans les deux genres et par tranche d’âge. 41,8% d’étudiants étaient inactifs, 56,8% actifs et 1,4% très actifs.

**Table 2:** Distribution des obèses, de la PASN et des diabétiques par tranches d’âge

	15-19 ans		20-24 ans		25-29 ans		30-35 ans	
	H	F	H	F	H	F	H	F
Effectif total	61	109	689	869	456	395	48	31
Sujets obèses (effectif)	1	5	6	32	6	39	1	5
Sujets à PASN (effectif)	9	7	125	63	84	38	8	3
Sujets diabétiques (effectif)	0	1	3	12	0	8	0	0

PASN: Pression artérielle supérieure à la normale, H: hommesF: femmes

**Table 3:** Niveau d'activité physique des étudiants

	15-19 ans		20-24 ans		25-29 ans		30-35		Total	
	H	F	H	F	H	F	H	F	H	F
Inactifs (effectif)	20	60	164	480	117	229	18	24	319	793
Actifs (effectif)	40	48	513	381	332	161	28	7	913	597
Très actifs (effectifs)	1	1	13	8	7	4	2	0	23	13

H: hommes F: femmes

L'APS était inversement corrélée à l’âge (r =-0,015; p < 0,0001) et au temps passé devant la télévision (r = -0,059; p = 0,002). L'IMC était corrélé au temps passé devant la télévision (r = 0,103; p < 0,0001). Parmi les participants, 1125 (42,3%) se sont déclarés consommateurs d'alcool et 16 (0,6%) fumeurs. Des associations positives ont été trouvées entre la consommation d alcool et respectivement et la PA (r = 0,469; p < 0,0001), la glycémie ≥ 1,26 g/L (r = 0,620; p < 0,0001) et l'obésité (r = 0,592; p < 0,0001). De même des associations positives ont été trouvées entre le tabagisme et respectivement la PA (r = 0,825; p < 0,0001), la glycémie ≥ 1,26 g/L (r = 0,979; p < 0,0001) et l obésité (r= 0,940; p < 0,0001).

## Discussion

Très peu ou pas d études ayant pour objectif le dépistage des MCV et portant spécifiquement sur les étudiants dans le contexte de pays pauvre ont été menées à notre connaissance. Comparées aux résultats obtenus sur la population générale dans une récente étude par Epacka et al. [19] dans la ville de Douala dans laquelle des sujets âgés de plus de 35 (plus nombreux) étaient inclus, les prévalences d'une PASN, du diabète et de l'obésité sont plus réduites dans la présente étude (respectivement 38,1%, 4,4% et 37% vs 12,7%, 0,9% et 3,6%). Cependant, les proportions des étudiants ayant une PASN et des obèses sont préoccupantes pour la moyenne d’âge de l’échantillon. Ceci peut s'expliquer par le taux élevé d'inactivité physique (42,4%) ainsi que des facteurs comportementaux déjà incriminés pas certains auteurs camerounais [20]. Les taux élevés de la consommation d'alcool et tabac bien qu'inférieurs à ceux de la population générale [20], témoignent d'une hygiène de vie chez les étudiants les exposant aux maladies cardiovasculaires. Une augmentation de la prévalence des facteurs de risque est à redouter si des mesures préventives comme la pratique des APS, la réduction de la consommation d'alcool et du tabac ne sont pas sérieusement prises en compte par les étudiants. La corrélation inverse retrouvée dans cette étude entre le temps passé devant la télé et l'activité physique concorde avec les travaux de Steffen et al [12] et Salmon et al [13]. Ce résultat signifiant une baisse de la pratique des APS en faveur de la télévision, permet également de comprendre la corrélation entre le temps passé devant la télévision et la présence des facteurs de risque cardiovasculaires (Lumeng et al. [11]). L'association entre les facteurs de risque et le niveau académique des étudiants pourrait refléter un effet de stress du fait que plus ces derniers progressent, plus les préoccupations et les charges académiques augmentent réduisant aussi le temps consacré à la pratique des APS. En effet, le rôle du stress est bien démontré dans la genèse des facteurs de risque cardio-vasculaire [18].

## Conclusion

Les maladies cardiovasculaires ainsi que les facteurs de risques sont présents en milieu estudiantin camerounais. Cette étude permet ainsi d'alerter les consciences sur les risques dus à l'inactivité physique et aux habitudes toxiques des étudiants. Une sensibilisation ciblée sur cette population devrait permettre de réduire la morbi-mortalité due aux maladies cardiovasculaires.

## References

[1] Beaglehole R, Yach D (2003). Globalisation and the Prevention and Control of Non-Communicable Disease: The Neglected Chronic Diseases of Adults. Lancet..

[2] Mbewu AD (2009). The burden of cardiovascular disease in sub-Saharan Africa. Summer..

[3] WHO (2005). Preventing Chronic Diseases: A Vital Investment.

[4] Paule P, Braem L, Mioulet D, Gil Jm, Theron A, Héno P, Fourcade L (2007). Insuffisance cardiaque d'origine non infectieuse en zone tropicale: Approche étiologique et principes thérapeutiques. Med Trop (Mars)..

[5] Bruckert E (2000). Activité physique et dyslipidémies. Le concours médical.

[6] Rieu M (1995). Rôle des activités physiques dans une politique de Santé publique. Bull Acad Natl Med.

[7] Mbanya JC, Minkoulou EM, Salah JN, Balkau B (1998). The prevalence of hypertension in rural and urban Cameroon. Int J Epidemiol..

[8] Chobanian AV, Bakris GL, Black HR, Cushman WC, Green LA, Izzo JL, Jones DW, Materson BJ, Oparil S, Wright JT, Roccella EJ (2003). The national high blood pressure education program coordinating commitee Seven report of the joint national commitee on prevention, detection, evaluation and treatment of high blood pressure. Hypertension..

[9] Ferdinand KC, Saunders E (2006). Hypertension-related morbidity and mortality in African Americans: Why we need to do better. J Clin Hypertens (Greenwich)..

[10] Lumeng JC, Rahnama S, Appugliese D, Kaciroti N, Bradley RH (2006). Television exposure and overweight risk in preschoolers. Arch Pediatr Adolesc Med..

[11] Steffen LM, Dai S, Fulton JE, Labarthe DR (2009). Overweight in children adolescents associated with TV viewing and parental weight Project heart beat!. Am J Prev Med..

[12] Salmon J, Timperio A, Telford A, Carver A, Crawford D (2005). Association of family environment with children's television viewing and with low level of physical activity. Obes Res..

[13] Eisenman JC, Bartee RT, Wang MQ (2002). Physical activity, TV viewing, and weight in US youth: 1999 Youth Risk Behavior Survey. Obes Res..

[14] World Health Statistics Annual (1995).

[15] Ricci et Gagnon (2009). Evaluation du niveau d'activité physique et de condition physique. http://www.santea.com.

[16] WHO (1995). Physical status: the use and interpretation of anthropometry. Report WHO Expert Committee.

[17] WHO (1999). World Health Organization- International Society of Hypertension. Guidelines for the management of hypertension. J Hypertens.

[18] Summary of technical report and recommendations in definition and diagnosis of diabetes mellitus and intermediate hyperglycemia (2006).

[19] Epacka Ewane M, Mandengue SH, Ahmadou G, Moumbe Tamba S, Dzudie A, Luma HN (2011). Dépistage des maladies cardiovasculaires et des facteurs de risque dans une cohorte de 270 Camerounais: Effets des activités physiques et sportives. Médecine des maladies Métaboliques..

[20] Awah PK, Kengne AP, Fezeu LK, Mbanya JC (2007). Perceived risk factors of cardiovascular diseases and diabetes in Cameroon. Health Education research..

[21] Chandola T, Brunner E, Marmot M (2006). Chronic stress at work 241 and the metabolic syndrome: prospective study. BMJ..

